# Heat Therapy for Musculoskeletal Pain Conditions: Actionable Suggestions for Pharmacists from a Panel of Experts

**DOI:** 10.3390/pharmacy13030063

**Published:** 2025-04-29

**Authors:** Flavia Nossa, Massimiliano Franco, Alberto Magni, Emanuela Raimondo, Giuseppe Ventriglia, Fabrizio Gervasoni

**Affiliations:** 1 Independent Researcher, Community Pharmacist, 24047 Treviglio, Italy; nf2529bg2012@pec.fofi.it; 2 SIMG (Italian College of General Practitioners and Primary Care), 50123 Florence, Italy; mass.franco@gmail.com (M.F.); magni.alberto@simg.it (A.M.); g.ventriglia@dag.it (G.V.); 3 Prosthetic Orthopedics and Hip Knee Reconstruction Unit, Humanitas Hospital, 20089 Rozzano, Italy; emanuela.raimondo@sanpiox.humanitas.it; 4 S.C. District Municipality 2, ASST Fatebenefratelli Sacco, 20157 Milan, Italy; 5 Industrial Engineering PhD Program, Industrial Engineering Technologies for Sports Medicine and Rehabilitation, University of Rome Tor Vergata, 00133 Rome, Italy

**Keywords:** heat therapy, musculoskeletal disorders, pain, pharmacist, competency, decisional algorithm

## Abstract

Musculoskeletal disorders represent one of the most pervasive health concerns that drive frequent medical consultations and pharmacy encounters. Community pharmacies are well placed to help address this demand as they are accessible settings for healthcare advice and support for patients with musculoskeletal disorders complaining of pain. Heat therapy stands as a valuable component of a multimodal approach to the management of musculoskeletal pain by virtue of multiple effects: pain relief, reduction of muscle spasms and stiffness, and enhanced muscle flexibility and range of motion. However, there is limited guidance on heat therapy use in routine practice, particularly on indications and contraindications, mode of application, and precautions. Such an educational gap has been documented among pharmacists. Therefore, it is paramount that pharmacists gain knowledge about when and how to effectively integrate superficial heat therapy with both pharmacological and physical therapy, to provide patients with a comprehensive, multimodal approach to alleviating musculoskeletal pain. A multidisciplinary panel of experts gathered to develop practical guidance on heat therapy-appropriate application in patients with musculoskeletal pain. In this work, we provide actionable suggestions to build pharmacists’ competency in managing musculoskeletal pain and empower them in effectively using heat therapy as a single therapeutic option or in combination with over-the-counter analgesics.

## 1. Introduction

Musculoskeletal (MSK) disorders encompass a wide range of conditions, affecting joints, bones, muscles, and tendons and characterized by pain, disability, and reduced quality of life (QoL). MSK disorders impose a significant health burden with 1.7 billion people affected worldwide and their prevalence is rapidly rising because of population growth and ageing [1,2]. MSK disorders account for up to one-third of general practice (GP) consultations [3] and are a very common reason for entering a community pharmacy to seek advice [4]. Given the increasing burden of MSK disorders among the general population, pharmacists’ proximity to the community could be key to optimizing MSK care. Community pharmacists can provide information about MSK pain self-management and have a crucial role in guiding patients toward rational pharmacotherapy or alternative pain management strategies, thus, ultimately, enabling a more individualized approach to MSK pain management [5].

Heat therapy (HT) stands as a valuable therapeutic option in the management of MSK pain by virtue of multiple effects: pain relief, reduction of muscle spasms and stiffness, and enhanced muscle flexibility and range of motion. Overall, these effects aid a fast resumption of normal functioning and participation in work/daily activities [6,7]. HT can be delivered in multiple forms including superficial heat pads or wraps, heat lamps, diathermy, and ultrasounds. Importantly, HT, in the form of superficial low-level heat wraps, can serve as an inexpensive treatment choice in both inpatient and outpatient settings as well as a suitable option for home therapy [8]. Despite the mounting evidence supporting HT clinical utility in MSK pain management as documented in both randomized multicenter clinical trials conducted in patients suffering from spinal (low back and neck), knee and wrist pain and meta-analysis and systematic reviews as well as in consensus papers [6,9,10,11,12,13,14], there is limited guidance on its use in routine practice, particularly on indications and contraindications, mode of application, and precautions. Such an educational gap has been documented across different healthcare professionals including pharmacists [4,14]. To date, the lack of an appropriate education may significantly hinder pharmacists in recommending superficial heat therapy (SHT), particularly through continuous low-level heat wrap application, to their patients suffering from MSK pain. Therefore, there is a pressing need to build pharmacists’ competency in managing MSK pain with HT with the final aim of enhancing their confidence in recommending it alone or in combination with analgesic medications.

To this end, a multidisciplinary panel of experts, encompassing one community pharmacist, two general practitioners (GPs), one physiatrist, and one orthopedic gathered in Milan (Italy) on 16 October 2024, to develop practical guidance on HT appropriate application in patients with MSK pain. The experts were selected based on their experience in SHT and, prior to the meeting, were asked to identify key topics that could be of interest to the everyday routine practice of pharmacists dealing with subjects suffering from MSK conditions. The authors identified the following themes: red flag identification, profiling of the patients who may seek help in the pharmacy, and differences between heat and cold application in terms of pain relief and integration of heat therapy with over-the-counter analgesic medications. During the in-person meeting, the authors developed the proposed algorithm and actionable suggestions. The aim of this work is to present the main insights from the panel discussion and provide actionable suggestions to empower pharmacists in effectively managing MSK pain with heat therapy.

## 2. Pharmacy Encounters: The Patient with a Localized MSK Pain Seeking Advice

In everyday pharmacy practice, pharmacists are frequently required to manage patients with MSK disorders who complain of pain, stiffness, limited range of joint motion, and motion restriction. Moreover, depending on the affected body area, patients may also report tenderness and limited mobility [15,16], the presence of cracking at the knee joint level [8] or shoulder pain when lifting the arm above the head or moving it forward. MSK disorders may arise because of a sedentary lifestyle, an incorrect posture, lifting heavy objects or bending forward in an awkward position, knee osteoarthritis, activities that load the knee in flexion, such as running or squatting as well as overhead activities in the workplace and in sports (e.g., volleyball, baseball, basketball). To date, MSK injuries frequently occur in sport with shoulder pain being one of the most common MSK complaints and regarded as extremely debilitating for athletes in overhead sports [17]. Overall, regardless of the pain location, patients suffering from MSK disorders report functioning issues, difficulties in participating in daily activities, emotional distress, and reduced QoL [18].

MSK pain is commonly treated with local heat applications (LHAs), and SHT, in the form of continuous low-level heat wraps, can be integrated into the treatment of MSK pain by different healthcare providers including pharmacists [19]. When dealing with MSK disorders and related pain, pharmacists are required to initially devote their time to patients’ information gathering and identification of red flags before counselling on using HT. This initial step may support pharmacists in choosing whether (a) to recommend considering HT, (b) to recommend considering HT but advise patients to see the doctor before use, or (c) to recommend avoiding HT because of the presence of red flags that would require referral to either a GP or other specialist care.

### 2.1. Red Flags’ Identification

Pharmacists are advised to first ask for the complaining patient’s age. SHT should not be used on children who are too young to be able to remove the wrap by themselves if they feel discomfort when unassisted. Thus, patients’ age < 12 years is regarded as a key red flag. Skin integrity is required for SHT [6]. Skin conditions such as contact dermatitis or eczema can be triggered by high temperatures and by low humidity, so dry heat therapy in particular can lead to flare-ups. The presence of bruises or signs of an early stage of an injury or of a trauma are also important red flags as heat application on open wounds or hematomas may increase blood flow to the wound and potentially increase bleeding. In addition, the early stage of an injury/trauma may suggest a potential ongoing inflammation which prevents the use of SHT. If one of the aforementioned conditions is met, pharmacists are advised to avoid counselling patients on using HT. Further contraindications to HT can be resumed as undiagnosed acute MSK injuries, inflammatory processes, actual or suspected bacterial infective or rheumatologic diseases in an active phase [8]. HT should be used with caution in patients with diabetes mellitus, multiple sclerosis, poor circulation, spinal cord injuries, and rheumatoid arthritis because it may cause disease progression, burns, skin ulceration, and increased inflammation [20]. Moreover, a history of serious conditions such as cancer should also be regarded as a relevant warning and demands referral to the practicing physician before using HT.

Importantly, patients with diabetes mellitus are characterized by impaired circulation or capillary fragility and may exhibit a decreased ability to sense heat, thus being more prone to irritation than healthy subjects. For this reason, pharmacists are advised to recommend patients to consult their doctors before using HT. Similarly, patients with heart disease, such as chronic heart failure, experience an impaired body’s ability to increase blood flow and sweat in response to heat; this weakened reaction can aggravate cardiac illnesses, even including hypertension. Therefore, patients with cardiovascular problems need to gradually bring their body temperature down after HT. In addition, cardiovascular patients taking anticoagulant therapies for secondary prevention could be considered ineligible for SHT, particularly those with fragile skin that is easily bruised. Overall, caution should be exercised with patients suffering from conditions where peripheral sensitivity may be altered such as multiple sclerosis, amyotrophic lateral sclerosis, spinal injuries, and diabetes [6]. Finally, pregnancy and breastfeeding are not a contraindication for using SHT; nevertheless, pharmacists should consider HT only in agreement with an authorized healthcare professional.

### 2.2. HT vs. Cold Therapy in MSK Disorders

Both HT and cold therapy are commonly employed to promote pain relief and healing and to restore function in patients with MSK disorders [21]. However, there is prevalent confusion among healthcare providers, including pharmacists, about which modality (heat vs. cold) to use and when to use it. To build pharmacists’ competency in managing MSK pain with HT, it is paramount to highlight the differences between these two non-pharmacological approaches and emphasize that the choice between HT and cold therapy should rely on the type of application and medical condition. HT improves muscle flexibility, increases blood flow and metabolism, and can contribute to the healing process. HT can be particularly beneficial for chronic nociceptive MSK pain, non-specific low back pain (LBP), mechanical pain, delayed onset muscle soreness (DOMS), strain, and sprain during the chronic phase of rehabilitation and should be applied once the oedematous phase of the healing process is over [6,21]. Evidence of HT efficacy in MSK pain conditions mostly stems from several prospective randomized multicenter studies, enrolling a number of patients ranging from fewer than 50 to almost 400 subjects depending on the anatomic area being investigated. Cold therapy reduces the blood flow, inflammation, muscle spasms, and metabolism. Cold application is mostly preferable for acute inflammatory pain conditions [21] and if the patients refer a recent trauma with inflammation and visible oedema (e.g., during the first 48 h to 72 h after an acute injury) or structural damage such as bone fractures, ligament tears, or muscle injury. Pharmacists may also encounter subjects complaining of pain after a rigorous workout and referring DOMS, which has been reported as being very common among both elite and novice athletes [22]. Both HT and cold therapy may promote recovery and relieve the pain of DOMS patients effectively within the first hour after exercise. However, as documented in a systematic review and meta-analysis of 32 randomized controlled trials, the pain-relieving effects following application of SHT are more long-lasting than cold therapy (over 24 h compared to within 24 h) [23] and appear to be of a greater extent [6,24]. Finally, when counselling patients with MSK disorders on using HT or cold therapy, the pharmacists should advise that an inappropriate use of HT or cold therapy may carry the risk of burns or skin ulceration or frostbite and superficial nerve damage or injury, respectively.

## 3. Guiding Pharmacists in Appropriately Selecting HT as a Single Therapeutic Option or in Combination with Over-the-Counter Analgesics

Following a careful assessment of patient history, of recent and long-term medication history and underlying or coexisting conditions as well as initial identification of potential red flags, pharmacists should focus on the patients’ symptom that determined the pharmacy encounter, namely the MSK pain. Pharmacists should be able to ask the patients with MSK disorders about their pain and learn more about the pain’s duration, onset, pain course, and characteristics. In this setting, it would be advisable to look for the circumstances of the onset of the pain, whether due to increased exertion, lifting, or injury, as it can provide a context to determine the underlying cause.

Table 1 illustrates potential questions pharmacists may ask the patients with MSK disorders entering their pharmacy to seek advice for pain relief. Such an approach may support pharmacists in obtaining plentiful and useful clues about their patients’ pain complaint.

Pharmacists are advised to refer the patient to a GP or specialists if the pain has lasted for more than one week and if the pain does not worsen on movement. SHT is indicated for the short-term relief of MSK pain. If the patients complain their pain has been present for more than one week, it is likely that further diagnostic evaluations are needed to both unveil the underlying cause and to develop an appropriate treatment plan. If the patients complain that their pain does not worsen on movement, pharmacists are encouraged to prompt an immediate referral to a GP or specialists as it may be a sign of a potential visceral origin of pain or the presence of systemic causes which demand immediate intervention from specialized healthcare providers.

SHT can be regarded as part of a multimodal approach to MSK pain management [6]. Therefore, it is paramount that pharmacists gain knowledge about when and how to effectively integrate SHT with both pharmacological and physical therapy, to provide patients with a comprehensive, multimodal approach to alleviating MSK pain. Mounting evidence supports the incorporation of SHT into multimodal strategies aimed at providing pain relief and restoring function in patients complaining of MSK pain. Implementing SHT into a multimodal treatment regimen for chronic LBP was documented to significantly improve muscular strength for both extension and rotation [9]. Moreover, in a randomized controlled study carried out on 92 patients with non-specific neck pain, combining SHT with ibuprofen was reported to effectively provide pain relief, a reduction in the disability index score and increased ROM in patients with neck pain undergoing physical therapy [13]. Overall, SHT can serve as a useful adjunct to both pharmacological and conventional physical therapy by virtue of improved pain control, a faster return to function, and greater compliance with home exercise. When gathering information about patient medication history, pharmacists are advised to ask the patients whether they are already taking pain medications or are currently undergoing physical exercise. If the patients are naïve to pain medication, pharmacists can consider dispensing SHT while advising the patients to refer to their GPs if no symptom improvement occurs by day 7. SHT can stand as a valuable option in comorbid and frail patients such as elderly patients who are already receiving several concomitant medications. Frailty and polypharmacy are very close in the elderly patient who is often burdened with concomitant conditions which should be taken into account when a new pain treatment needs to be considered. Of note, in the elderly the pharmacological interactions between current chronic therapies and new treatments could lead to safety issues. In this scenario, the availability of options endowed with a limited risk of drug–drug interaction would be preferable. Thus, SHT can stand as a valuable option in elderly patients who are already receiving several concomitant medications pending further GP consultation in the presence of conditions such as diabetes, multiple sclerosis, rheumatoid arthritis, poor circulation, and cardiovascular diseases. However, if the patient is older than 55, pharmacists should advise against night-time use of SHT. If the patients are already taking analgesics, SHT can be incorporated in the current treatment regimen and may also help reduce the dosage of pain medications. If the patients are taking topical pain medications, pharmacists should advise patients to not use SHT with pain rubs, medicated lotions, creams, and ointments. If no symptom improvement occurs after 7 days of combined SHT and over-the-counter analgesics, pharmacists should always advise patients to contact their GPs for further evaluation.

**Figure 1:**
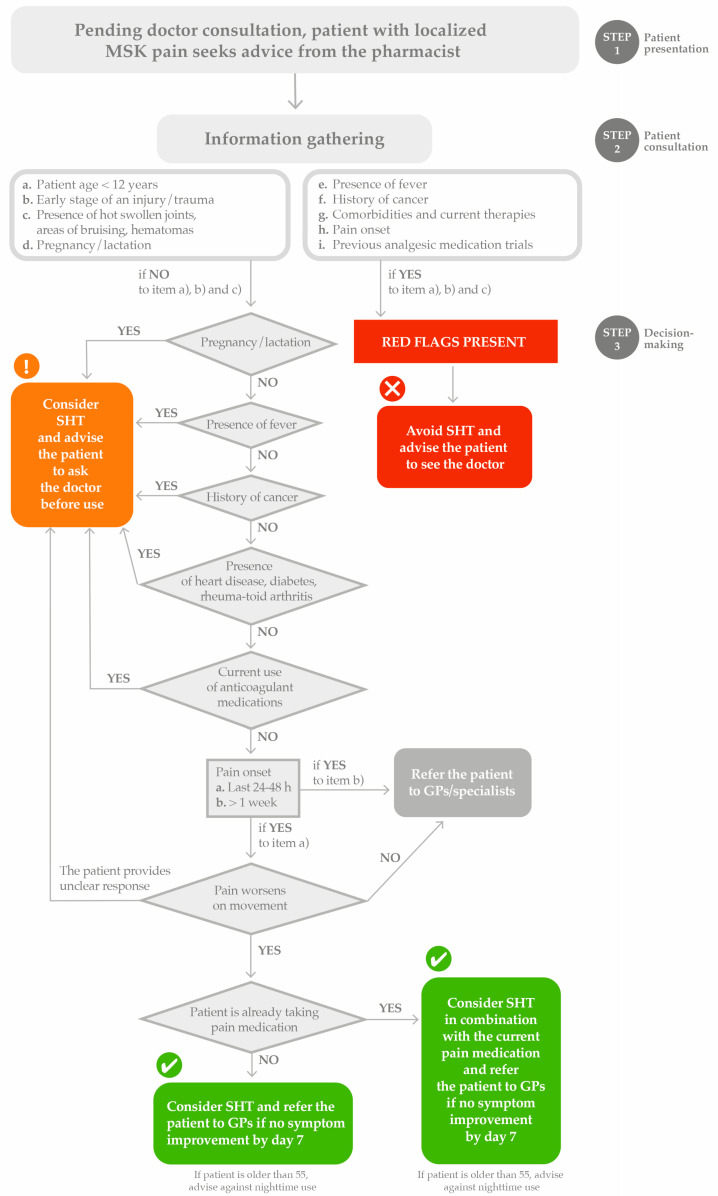
Decisional algorithm for MSK pain management in pharmacy. Heat therapy as a standalone option or in combination with analgesic medications. GP, general practitioner; SHT, superficial heat therapy.

A user-friendly decisional algorithm is provided in Figure 1 to aid pharmacists in counselling patients with MSK pain seeking advice pending a doctor consultation.

## 4. Conclusions and Perspectives

MSK disorders represent one of the most pervasive health concerns that drive frequent medical consultations and pharmacy encounters. The increasing ageing of the population and the rising prevalence of sedentary lifestyle-related disorders significantly contribute to the burdensome impact of MSK disorders in primary care practice, thus leading to a greater demand for medical consultation. Community pharmacies are well placed to help address this demand as they are accessible settings for healthcare advice and support for patients with MSK disorders complaining of pain [3,4]. Over the past few decades, pharmacies have been growing fundamental structures of the healthcare system which are expanding their activities by shifting from product-oriented to patient-oriented services, thus increasingly integrating into long-term condition care pathways. Therefore, pharmacies can be crucial for optimal and sustainable healthcare delivery, especially at a time of increasing demand [25].

MSK pain is prevalent and, if left untreated or inadequately controlled, can develop into chronic pain syndromes that can be challenging to manage. Regardless of the etiology of MSK pain, it is paramount to initiate pain treatment as early as possible and to control pain over time by targeting the underlying mechanisms with the final aim of preserving MSK function. Therefore, pending a doctor consultation, which may require a few days to be scheduled, patients may initially seek advice in the pharmacy. With so many non-pharmacological interventions and over-the-counter analgesics available, it can be confusing for patients looking to self-manage their pain symptoms. In this scenario, pharmacists should be engaged to effectively counsel patients with MSK pain and provide pharmaceutical advice on which pain-relieving interventions to choose to institute pain therapy as early as possible. However, pharmacists often face barriers such as time constraints and insufficient training [26]. In a recent survey, 8 in 10 pharmacists declared the need to address significant knowledge gaps in pain management [5] and such educational needs appear substantial in the management of MSK pain as pharmacists perceive themselves as not properly trained to identify, assess, and manage MSK pain [4]. Moreover, limited guidance on the use of non-pharmacological approaches to MSK pain, including SHT, has been recently documented among pharmacists [4]. Thus, there is the need to build pharmacists’ competency in leveraging on SHT to provide advice for self MSK pain management.

Pharmacists need to be supported to undertake appropriate training so they can better support patients with MSK disorders. To this end, postgraduate training and information tools could be helpful. In everyday practice, pharmacists can benefit from hands-on tools and decisional algorithms specifically developed to provide practical guidance on SHT’s proper application, highlighting specific potentialities as well as contraindications. The decisional algorithm shown in Figure 1 aims to aid pharmacists in counselling patients with MSK pain on using SHT pending a doctor consultation by guiding step by step the pharmacists in red flags identification, patient assessment, and the treatment decision. During this manuscript preparation, the authors were made aware, via an online search, that the International Pharmaceutical Federation (FIP) had just released a guide to support community pharmacists in the dispensing of HT in the management of MSK pain [27]. The actionable suggestions (Table 2) provided in our work are fully in line with the latest FIP recommendations on HT in MSK disorders and complement them by providing a user-friendly workflow to follow by the pharmacists along with a list of patient-friendly questions to ask the patients to attain comprehensive information gathering.

In line with our work, a recent position paper shared HT decisional algorithms for anatomical sites deemed of particular interest regarding a potential response to exogenous SHT, namely the back, neck, shoulder, and knee [28], thus further supporting the ongoing educational efforts put in play to build and maintain the competency of healthcare providers in managing MSK pain with SHT. Future efforts should be made to develop appropriate referral pathways/protocols so that pharmacists know what is within their remit and when to refer as well as initiatives fostering collaboration with GPs and/or specialists including orthopedics and physiatrists and knowledge sharing to enhance pharmacists’ confidence in the management of MSK pain.

Pharmacist-delivered interventions may hold great potential in advancing pain management practices with broader implications for healthcare delivery. Integrating pharmacists into pain management strategies improves patient outcomes and supports the highly advocated transformative shift towards a more holistic, patient-centered care approach [29,30]. Building competency in managing MSK pain, including using SHT, may provide two-fold advantages to MSK patient care. First, patients may receive pain relief pending a doctor consultation and, in the presence of symptom improvement, would likely not need to see the doctors, thus sparing the latter unnecessary patient visits. Second, even if the pain relief attained with SHT would not have been sufficient to avoid a secondary referral, patients’ pain condition could be less challenging to manage by the specialists compared to that of patients untreated or postponing medical consultation until the pain is severe.

In summary, empowering pharmacists in effectively managing MSK pain with SHT and addressing knowledge gaps related to the use of SHT can be used to both optimize MSK care provision and improve MSK patients’ journey.

## Figures and Tables

**Table 1:** Pharmacist-led MSK patient pain assessment: suggested questions.

Pharmacist-Led Patient Pain Assessment	Questions
Duration of the pain	When it started and how long it has lasted? Have you been suffering for more than a week?
How the pain started	What were you doing when the pain started?
Characteristics of the pain	What does your pain feel like? Point to where it hurts the most. Where does your pain go from there? What makes your pain better or worse? Does the pain get worse during night-time?
Pain course	Would you consider yourself constantly in pain?
Pain severity	Please rate your pain by selecting a number between 0 and 10 that best describes your pain at its worst in the last 24 h
Current pain management	What treatments or medications, if any, are you receiving for your pain?

**Table 2:** Experts’ actionable suggestions.

Given the increasing burden of MSK disorders among the general population, pharmacists’ proximity to the community should be better exploited to optimize MSK care provision.
Adequate training and education on MSK pain management, including the use of non-pharmacological approaches, including SHT, would empower pharmacists to provide the patients with well-informed advice and appropriate interventions.
A hands-on approach to the management of MSK pain is desirable to build and maintain pharmacists’ competency in counselling patients on using SHT as a valuable non-pharmacological option either alone or in combination with over-the-counter pain medications.
Establishing collaborative relationships with other healthcare professionals, such as GPs, physiatrists, orthopedics, and physiotherapists may facilitate a multidisciplinary approach to patient care and promote a full integration of the pharmacists into MSK pain care pathways.

## Data Availability

The original contributions presented in this study are included in the article. Further inquiries can be directed to the corresponding author.

## Abbreviations

The following abbreviations are used in this manuscript:

DOMS	Delayed Onset Muscle Soreness
GP	General Practitioner
HT	Heat Therapy
LBP	Low Back Pain
LHA	Local Heat Application
MSK	Musculoskeletal
QoL	Quality of Life
ROM	Range of Motion
SHT	Superficial Heat Therapy

## References

[1] Blyth F.M., Briggs A.M., Schneider C.H., Hoy D.G., March L.M. (2019). The global burden of musculoskeletal pain—Where to from here?. Am. J. Public Health.

[2] World Health Organization Musculoskeletal Conditions. https://www.who.int/news-room/fact-sheets/detail/musculoskeletal-conditions.

[3] Margham T. (2011). Musculoskeletal disorders: Time for joint action in primary care. Br. J. Gen. Pract..

[4] International Pharmaceutical Federation (FIP) (2024). Managing Musculoskeletal Pain in the Community Pharmacy: Report from an International Insight Board.

[5] Mujtaba S.H., Gazerani P. (2024). Exploring the Role of Community Pharmacists in Pain Management: Enablers and Challenges. Pharmacy.

[6] Lubrano E., Mazas P.F., Freiwald J., Kruger K., Grattagliano I., Mur E., Silva R.Q., Maruri G.R., de Medeiros L.S. (2023). An International Multidisciplinary Delphi-Based Consensus on Heat Therapy in Musculoskeletal Pain. Pain Ther..

[7] Clijsen R., Stoop R., Hohenauer E., Aerenhouts D., Clarys P., Deflorin C., Taeymans J. (2022). Local Heat Applications as a Treatment of Physical and Functional Parameters in Acute and Chronic Musculoskeletal Disorders or Pain. Arch. Phys. Med. Rehabil..

[8] Rossi R. (2024). Heat therapy for different knee diseases: Expert opinion. Front. Rehabil. Sci..

[9] Freiwald J., Magni A., Fanlo-Mazas P., Paulino E., de Medeiros L.S., Moretti B., Schleip R., Solarino G. (2021). A Role for Superficial Heat Therapy in the Management of Non-Specific, Mild-to-Moderate Low Back Pain in Current Clinical Practice: A Narrative Review. Life.

[10] Nadler S.F., Steiner D.J., Erasala G.N., Hengehold D.A., Hinkle R.T., Goodale M.B., Beln S.B., Weingand K.W. (2022). Continuous low-level heat wrap therapy provides more efficacy than Ibuprofen and acetaminophen for acute low back pain. Spine.

[11] Petrofsky J., Laymon M., Alshammari F., Khowailed I.A., Lee H. (2015). Continuous Low Level Heat Wraps; Faster Healing and Pain Relief during Rehabilitation for Back, Knee and Neck Injuries. Prev. Med..

[12] Petrofsky J., Laymon M.S., Alshammari F.S., Lee H. (2016). Use of low level of continuous heat as an adjunct to physical therapy improves knee pain recovery and the compliance for home exercise in patients with chronic knee pain: A randomized controlled trial. J. Strength Cond. Res..

[13] Petrofsky J., Laymon M., Alshammari F., Khowailed I.A., Lee H. (2017). Use of low level of continuous heat and ibuprofen as an adjuct to physical therapy improves pain relief, range of motion and the compliance to home exercise in patients with nonspecific neck pain: A randomized controlled trial. J. Back Musculoskelet. Rehabil..

[14] Hotfiel T., Fanlo-Mazas P., Malo-Urries M., Paulino E., de Medeiros L.S., Blondett M., Vetrano M., Freiwald J. (2024). Importance of heat therapy in the treatment of pain in the daily clinical practice. J. Bodyw. Mov. Ther..

[15] GBD 2021 Neck Pain Collaborators (2024). Global, regional, and national burden of neck pain, 1990-2020, and projections to 2050: A systematic analysis of the Global Burden of Disease Study 2021. Lancet Rheumatol..

[16] Moretti A., Menna F., Aulicino M., Paoletta M., Liguori S., Iolascon G. (2020). Characterization of Home Working Population during COVID-19 Emergency: A Cross-Sectional Analysis. Int. J. Environ. Res. Public Health.

[17] Hoppe M.W., Brochhagen J., Tischer T., Beitzel K., Seil R., Grim C. (2022). Risk factors and prevention strategies for shoulder injuries in overhead sports: An updated systematic review. J. Exp. Orthop..

[18] Giua C., Minghetti P., Gandolini G., Rocco P., Arancio E., Bevacqua T., Floris N., Keber E., Sgcp, Musazzi U.M. (2020). Community Pharmacist’s Role in Detecting Low Back Pain, and Patient Attitudes-A Cross-Sectional Observational Study in Italian Community Pharmacies. Int. J. Environ. Res. Public Health.

[19] Ventriglia G., Gervasoni F., Franco M., Magni A., Panico G., Iolascon G. (2023). Musculoskeletal Pain Management and Thermotherapy: An Exploratory Analysis of Italian Physicians’ Attitude, Beliefs, and Prescribing Habits. J. Pain Res..

[20] Nadler S.F., Weingand K., Kruse R.J. (2004). The physiologic basis and clinical applications of cryotherapy and thermotherapy for the pain practitioner. Pain Physician.

[21] Malanga G.A., Yan N., Stark J. (2015). Mechanisms and efficacy of heat and cold therapies for musculoskeletal injury. Postgrad. Med..

[22] Cheung K., Hume P., Maxwell L. (2003). Delayed onset muscle soreness: Treatment strategies and performance factors. Sports Med..

[23] Wang Y., Li S., Zhang Y., Chen Y., Yan F., Han L., Ma Y. (2021). Heat and cold therapy reduce pain in patients with delayed onset muscle soreness: A systematic review and meta-analysis of 32 randomized controlled trials. Phys. Ther. Sport.

[24] Petrofsky J.S., Khowailed I.A., Lee H., Berk L., Bains G.S., Akerkar S., Shah J., Al-Dabbak F., Laymon M.S. (2015). Cold vs. heat after exercise-is there a clear winner for muscle soreness. J. Strength Cond. Res..

[25] Tzortziou Brown V., Underwood M., Westwood O.M., Morrissey D. (2019). Improving the management of musculoskeletal conditions: Can an alternative approach to referral management underpinned by quality improvement and behavioural change theories offer a solution and a better patient experience? A mixed-methods study. BMJ Open.

[26] Barbee J., Chessher J., Greenlee M. (2015). Pain Management: The Pharmacist’s evolving role. Pharm. Times.

[27] International Pharmaceutical Federation (FIP) (2024). Using Heat Therapy for the Management of Musculoskeletal Pain: Guidance for Pharmacists.

[28] Ventriglia G., Franco M., Magni A., Gervasoni F. (2024). Treatment Algorithms for Continuous Low-Level Heat Wrap Therapy for the Management of Musculoskeletal Pain: An Italian Position Paper. J. Pain Res..

[29] Piquer-Martinez C., Urionagüena A., Benrimoj S.I., Calvo B., Martinez-Martinez F., Fernandez-llimos F., Garcia-Cardenas V., Gastelurrutia M.A. (2022). Integration of community pharmacy in primary health care: The challenge. Res. Soc. Adm. Pharm..

[30] Shrestha S., Iqbal A., Teoh S.L., Khanal S., Gan S.H., Lee S.W.H., Paudyal V. (2024). Impact of pharmacist-delivered interventions on pain-related outcomes: An umbrella review of systematic reviews and meta-analyses. Res. Soc. Adm. Pharm..

